# Water ingestion decreases cardiac workload time-dependent in healthy adults with no effect of gender

**DOI:** 10.1038/s41598-017-08446-4

**Published:** 2017-08-11

**Authors:** Cathriona Rosemary Monnard, Erik Konrad Grasser

**Affiliations:** Department of Medicine/Physiology, University of Fribourg, Chemin du Musée 5, 1700 Fribourg, Switzerland

## Abstract

Ingestion of water entails a variety of cardiovascular responses. However, the precise effect remains elusive. We aimed to determine in healthy adults the effect of water on cardiac workload and to investigate potential gender differences. We pooled data from two controlled studies where blood pressure (BP) and heart rate (HR) were continuously recorded before and after the ingestion of 355 mL of tap water. Additionally, we calculated double product by multiplying systolic BP with HR and evaluated spectral parameters referring to vagal tone. All parameters were investigated for potential differences based on gender. In response to water, HR, systolic BP, and double product decreased significantly during the first 30 min. However, these effects were attenuated for HR and double product and even abolished for systolic BP over the subsequent 30 min. Over the entire post-drink period (60 min), decreases in HR and double product (all *P* < 0.05) were observed. Spectral markers for vagal tone increased with the on-set of the water drink and remained elevated until the end (*P* < 0.005). No significant gender difference in cardiac workload parameters was observed. We provide evidence that drinking water decreases, in a time-dependent fashion, cardiac workload and that these responses appear not to be influenced by gender.

## Introduction

Water consumption induces a number of physiological alterations in the human cardiovascular system^1–4^, which can be best explained by its impact on the autonomic nervous system^5–7^. In this context, Jordan *et al*. observed a potent effect of water on blood pressure (BP) in patients with autonomic failure and attributed this finding to an activation of the sympathetic nervous system^5^. While these pressor effects of water are observed to a lesser degree in older subjects^5, 8^, such a pressor response was found to be absent in healthy and young subjects^4–7^. One potential explanation for the lack of BP changes in healthy adults in response to water drinking could be due to a simultaneous activation of the sympathetic and parasympathetic nervous system, thereby one counteracting the other^6^.

On the one hand, increasing BP augments the likelihood of severe cardiovascular events^9^, and a mere reduction of 2 mmHg in systolic BP is sufficient to decrease stroke mortality by 10%^9^. On the other hand, resting heart rate is known to affect the myocardial function^10^, at which elevated resting heart rate puts people at a higher risk for cardiovascular diseases^11, 12^. In this context, a simple product of systolic BP and heart rate, known as the rate pressure double product, is considered as a predictor of myocardial oxygen consumption^13, 14^ with, however, mixed results pertaining to its prognostic value for cardiovascular disease risk stratification^15, 16^.

Recently, evidence arose that ingestion of water decreases heart rate and rate pressure double product, thereby decreasing cardiac workload in a healthy subpopulation^4^, however, this finding is currently under debate^17, 18^. Moreover, despite a lack of clarity on the precise role of water on the cardiovascular system, researchers have begun to investigate whether gender differences exist in the response to water ingestion. To the best of our knowledge, there is only one study, which investigated potential gender differences in the response to water ingestion^19^.

Therefore, we pooled retrospectively 45 (22 women) healthy adults from two recent randomized controlled crossover studies where BP and heart rate were continuously recorded before and after the ingestion of 355 mL of tap water^20, 21^ in order to determine the effect of water on cardiac workload and to investigate potential gender differences.

## Material and Methods

We conducted a retrospective analysis of data from two recently published experiments, which were carried out in our laboratory^20, 21^. A statistical comparison between the two cohorts revealed no significant difference for age, weight, height or body mass index (all *P* > 0.05, combined and separated by gender), moreover, no significant difference between baseline measured heart rate, systolic BP, diastolic BP, stroke volume or the natural logarithmic transformation of the high frequency component of RR interval was observed between the two study groups (all *P* > 0.05, combined and separated by gender). Hence, we included and pooled data of a total of forty-five (23 male; 22 women) healthy subjects. Herein, a concise description of the methodology is provided; a more detailed description of the study methods can be found in the original publications^20, 21^. All analysis were performed strictly anonymized in a coded form.

In short, all participants fasted for ≥12 h and abstained from alcohol, smoking and caffeine, as well as from vigorous exercise for 24 h before each test and were advised not to change their diet between tests. Subjects with preexisting illnesses or on medication, which could take an impact on the study outcome, were not allowed to participate. Both studies were conducted according to the guidelines laid down in the Declaration of Helsinki, and the joint ethical committee of the States of Jura, Fribourg and Neuchâtel approved all procedures involving human subjects. All subjects provided written informed consent prior to the start of their studies.

After a period of stability (approximately 30 min), baseline recordings were taken for at least 20 min, which was followed by the ingestion of 355 ml tap water at room temperature over a period of 4 minute in order to ensure a convenient pace. Post-drink recordings were carried out and data for the first 60 min were pooled since both of our previous studies had similar protocols during the first 80 minutes with the peak hemodynamic response (with regard to the energy drink) reached around 60 to 80 min. Based on a previous publication where the hemodynamic impact of water temperature was investigated^4^, we decided on a 60 min lasting post-drink observational period.

Cardiovascular (systolic BP, diastolic BP, and stroke volume) and electrocardiographic (RR-intervals) recordings were performed beat-to-beat using a Task Force Monitor (*CNSystems*, Medizintechnik, Graz, Austria) and data were sampled at 1000 Hz. Heart rate was calculated from the appropriate RR-interval, cardiac output was computed as the product of stroke volume and heart rate, and total peripheral resistance was calculated as mean BP/cardiac output. We calculated mean BP from systolic and diastolic BP by the formula: mean BP equals diastolic BP + 1/3 (systolic BP – diastolic BP). Finally, rate pressure double product was calculated by systolic BP x heart rate.

High frequency (0.17–0.40 Hz) power components of RR-intervals (HF_RRI) were evaluated and given in absolute values (ms^2^). Keeping in mind the limitations^22^, we used changes in the high frequency range of heart rate variability to assess a surrogate of parasympathetic activity because HF_RRI is suggested to mediate parasympathetic nerve modulation^23, 24^. Powers of HF_RRI were analyzed after natural logarithmic transformation (HF_RRI_LN)^4^. Baroreflex sensitivity was determined from spontaneous fluctuations in BP and cardiac interval using the sequence technique^25^.

The index of contractility reflects the aortic peak flow and it is the maximum impedance changes (ΔZ/Δt_max_) normalized to the ground impedance Z_0_^26^.

Values of heart rate, systolic BP, diastolic BP, stroke volume, rate pressure double product, cardiac output, total peripheral resistance, index of contractility, HR_RRI_LN, and baroreflex sensitivity were averaged every 10 min during the baseline period and during the subsequent 60-min post-drink observation period.

**Figure 1:**
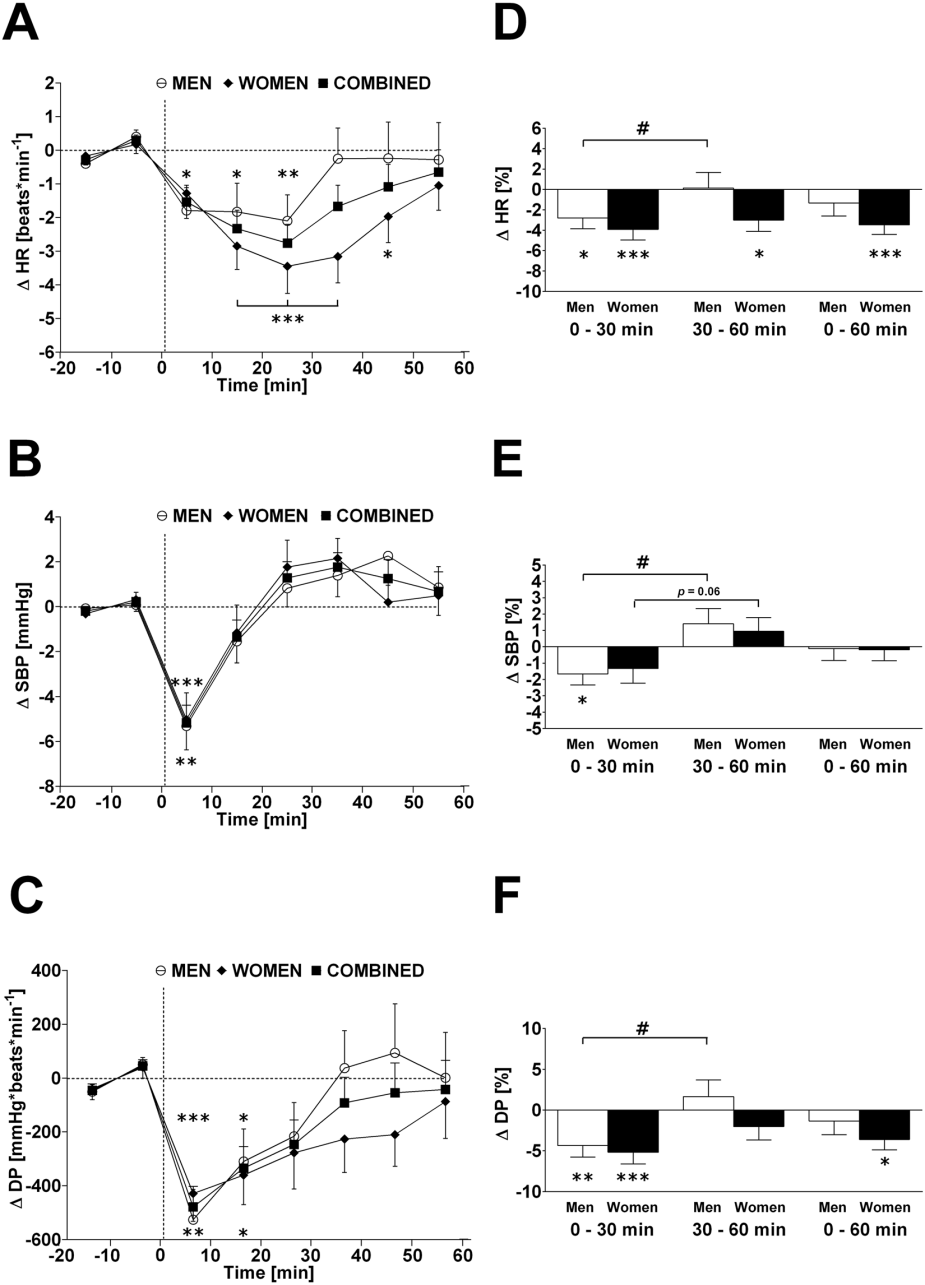
Left panels A–C: Time course of changes (Δ) from baseline in heart rate (HR), systolic blood pressure (SBP) and double product (DP), respectively. Open circle (○) = men; closed diamond (♦) = women; closed square (■) = combined men and women. Right panels D, E, F represent mean percentage changes in HR, SBP and DP relative to baseline. Time 0 denotes four minutes after the water drink with calculating a subsequent average over the following 30 minutes, 30 to 60 minutes, and 0 to 60 minutes post-drink, respectively, which were subtracted from each baseline level and presented as deltas. *P < 0.05, **P < 0.01 and ***P < 0.005 statistically significant differences over time from baseline values (left and right panels), ^**#**^ *P* < 0.005 statistically significant difference between 0–30 min and 30–60 min post-drink period (right panel). All values are reported as means ± SEM.

**Figure 2:**
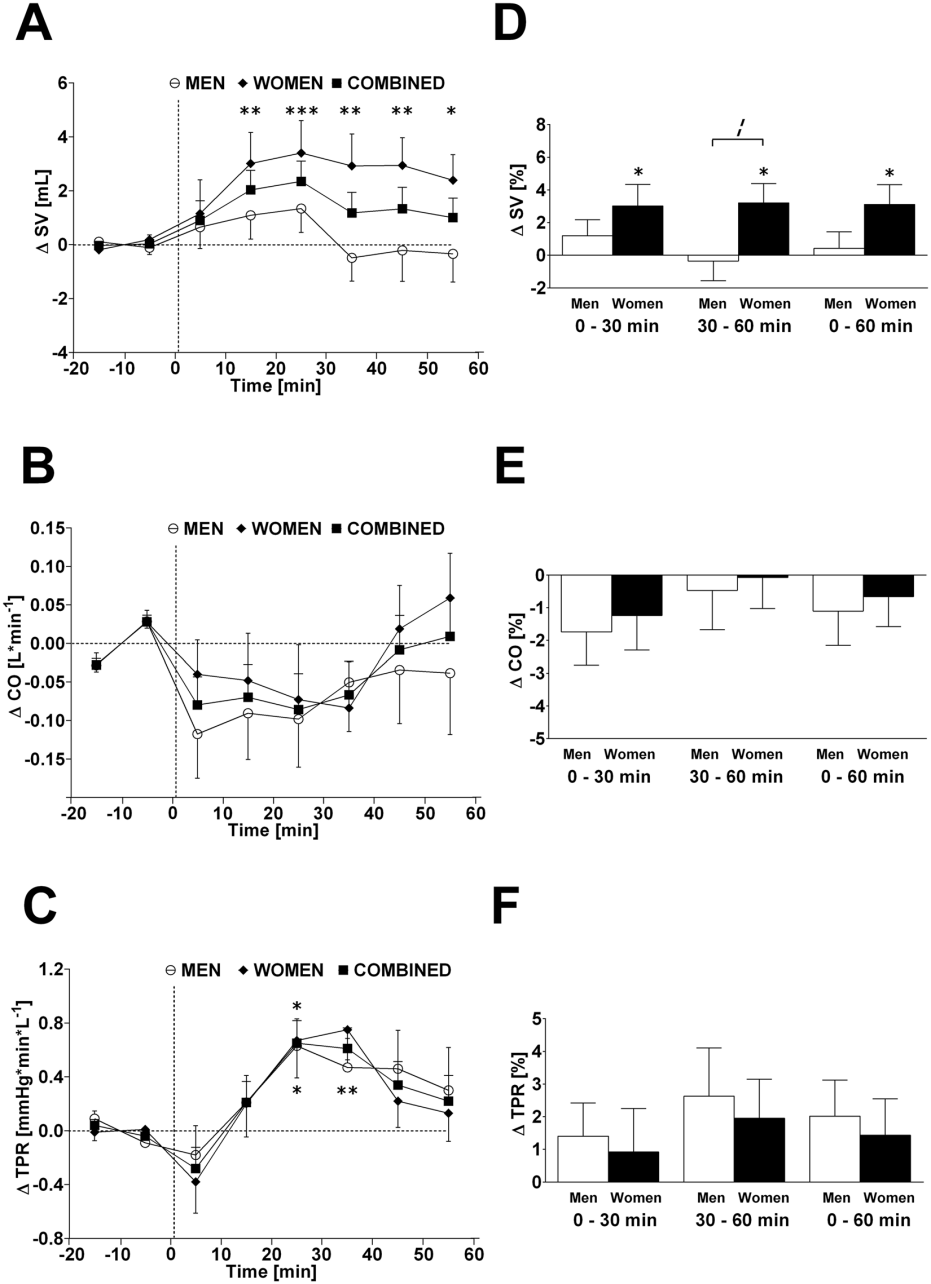
Left panels A–C: Time course of changes (Δ) from baseline in stroke volume (SV), cardiac output (CO) and total peripheral resistance (TPR), respectively. Open circle (○) = men; closed diamond (♦) = women; closed square (■) = combined men and women. Right panels D–F represent mean percentage changes in SV, CO and TPR relative to baseline. Time 0 denotes four minutes after the water drink with calculating a subsequent average over the following 30 minutes, 30 to 60 minutes, and 0 to 60 minutes post-drink, respectively, which were subtracted from each baseline level and presented as deltas. *P < 0.05, **P < 0.01 and ***P < 0.005 statistically significant differences over time from baseline values (left and right panels). ^***¦***^ *P* < 0.05 statistically significant difference between men and women at 30–60 min post-drink (right panel). All values are reported as means ± SEM.

**Figure 3:**
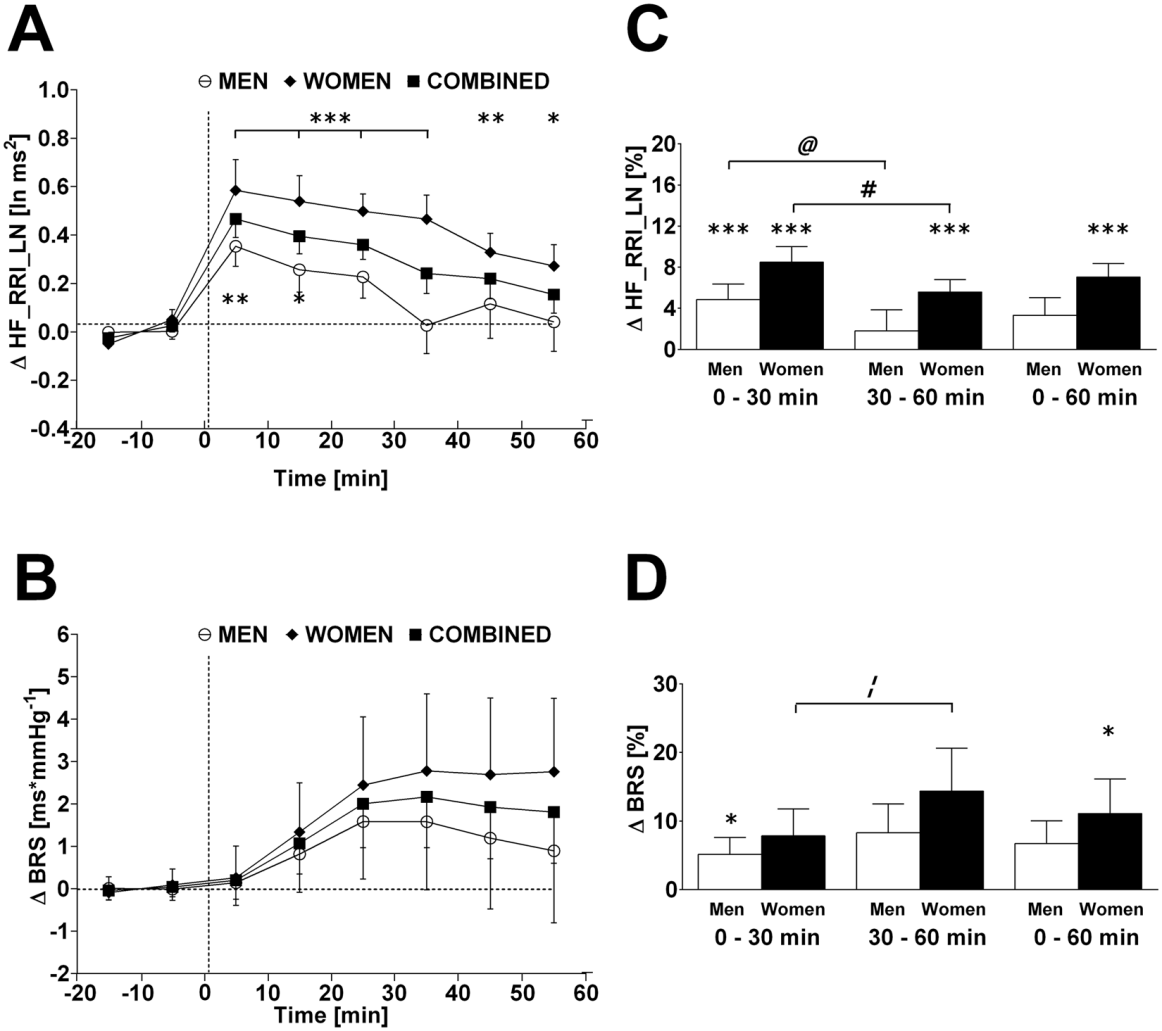
Left panels A and B: Time course of changes (Δ) from baseline in high frequency component of RR-interval (HF_RRI_LN) and baroreflex sensitivity (BRS), respectively. Open circle (○) = men; closed diamond (♦) = women; closed square (■) = combined men and women. Right panels C and D represent mean percentage changes in HF_RRI_LN and BRS relative to baseline. Time 0 denotes four minutes after the water drink with calculating a subsequent average over the following 30 minutes, 30 to 60 minutes, and 0 to 60 minutes post-drink, respectively, which were subtracted from each baseline level and presented as deltas. *P < 0.05, **P < 0.01 and ***P < 0.005 statistically significant differences over time from baseline values (left and right panels). ^**#**^ *P* < 0.005, ^***@***^ *P* < 0.01, and ^***¦***^ *P* < 0.05 statistically significant difference between 0–30 min and 30–60 min post-drink periods (right panel). All values are reported as means ± SEM.

**Figure 4:**
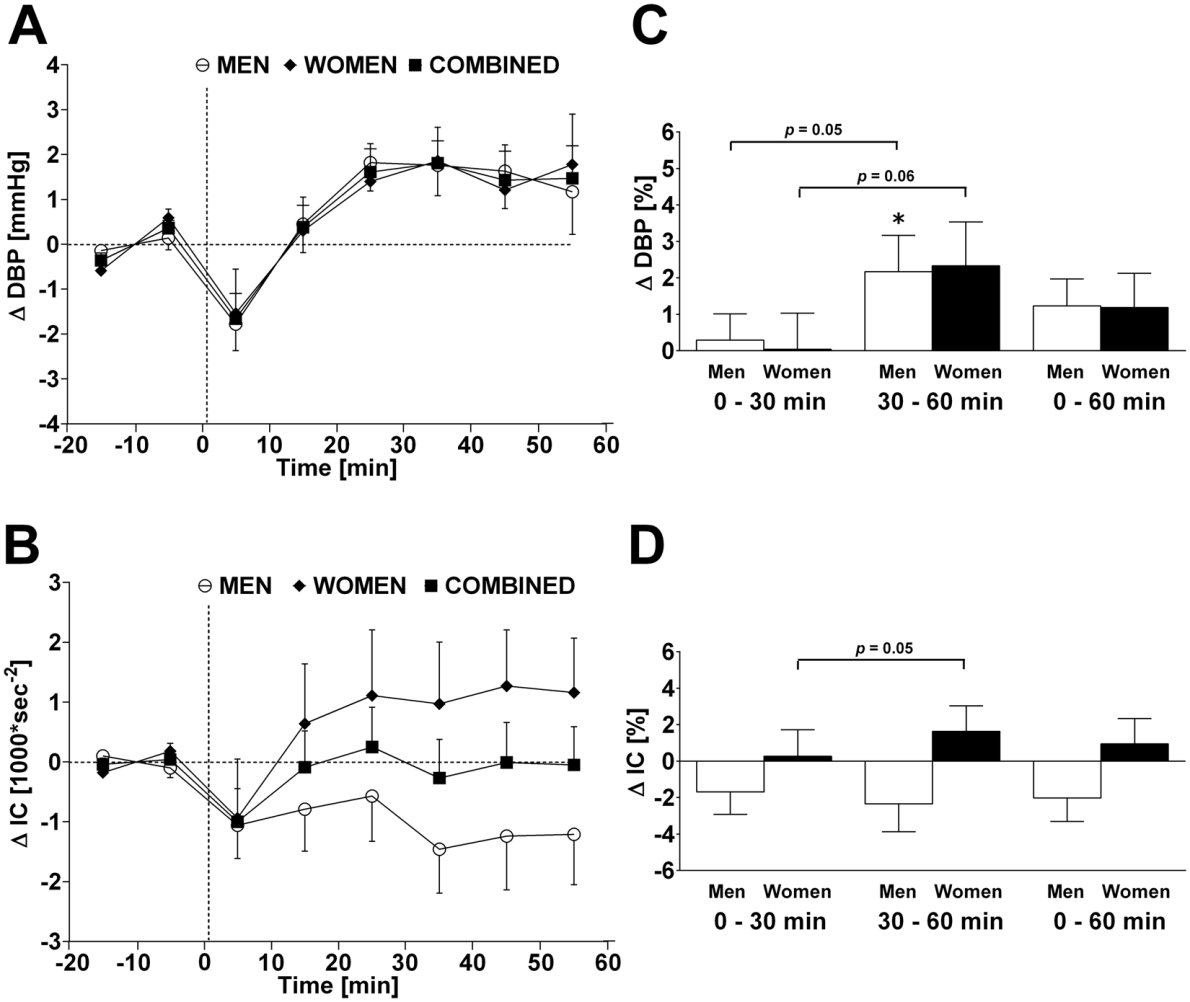
Left panels A and B: Time course of changes (Δ) from baseline in diastolic blood pressure (DBP) and index of contractility (IC), respectively. Open circle (○) = men; closed diamond (♦) = women; closed square (■) = combined men and women. Right panels C and D represent mean percentage changes in DBP and IC relative to baseline. Time 0 denotes four minutes after the water drink with calculating a subsequent average over the following 30 minutes, 30 to 60 minutes, and 0 to 60 minutes post-drink, respectively, which were subtracted from each baseline level and presented as deltas. *P < 0.05 statistically significant difference over time from baseline values (right panel). All values are reported as means ± SEM.

Data were analyzed using Statistix software (Version 8, Tallahassee, Florida, USA). Baseline data were analyzed using an unpaired student’s t-test where values were compared between men and women (Table 1). Repeated-measures *ANOVA* with *Dunnett*’s multiple comparison post hoc testing or the *Friedman* test with *Dunns* post hoc testing was used to test for changes over time from baseline level (Figs 1–4, left panels). Paired student’s t tests were performed to explore post-water changes (Table 2 and Figs 1–4, right panels). Statistical significance was set at a level of *P* < 0.05 and data are presented as mean ± standard error of the means.

**Table 1:** Baseline anthropometric- and cardiovascular parameters for 45 (22 women) young and healthy subjects.

Variable	Baseline all Mean ± SEM	Baseline Men Mean ± SEM	Baseline Women Mean ± SEM
Age, years	22.3 ± 0.4	23.2 ± 0.7*	21.4 ± 0.3
Height, cm	173 ± 1	179 ± 1***	167 ± 1
Weight, kg	68 ± 2	61 ± 1***	75 ± 2
Body-Mass-Index, kg*m^−2^	22.6 ± 0.4	21.8 ± 0.5*	23.4 ± 0.6
Heart rate, beats*min^−1^	62 ± 1	61 ± 2	63 ± 2
Systolic BP, mmHg	115 ± 1	119 ± 2***	111 ± 1
Diastolic BP, mmHg	74 ± 1	76 ± 1***	72 ± 1
Stroke volume, mL	83 ± 2	83 ± 2	83 ± 2
Double product, mmHg*beats*min^−1^	7108 ± 181	7286 ± 304	6922 ± 189
Cardiac output, L*min^−1^	5.1 ± 0.1	5.0 ± 0.1	5.2 ± 0.2
Total peripheral resistance, mmHg*min*L^−1^	17.5 ± 0.4	18.2 ± 0.4*	16.6 ± 0.6
Index of contractility, 1000*s^−2^	57 ± 1	50 ± 2***	64 ± 2
High-frequency component of RRI, ln ms^2^	6.8 ± 0.2	6.7 ± 0.2	7.0 ± 0.2
Baroreflex sensitivity, ms*mmHg^−1^	28.9 ± 2.0	27.0 ± 2.6	30.9 ± 3.1

All: Men and women combined; BP: blood pressure; RRI: RR-interval; ln: natural logarithm; statistical analysis was performed using an unpaired student’s *t*-Test where baseline values were compared between men and women. * *P* < 0.05 and ***P < 0.005 statistical significant differences between men and women.

**Table 2:** Post-water cardiovascular- and autonomic responses relative to baseline values and presented as a delta (Δ).

	Δ 0–30 min post-drink	Δ 30–60 min post-drink	Δ 0–60 min post-drink
Heart rate, beats*min^−1^	−2.2 ± 0.5***	−1.1 ± 0.6^@^	−1.7 ± 0.5***
% change	−3.3 ± 0.7***	−1.4 ± 1.0^#^	−2.4 ± 0.8**
Systolic BP, mmHg	−1.7 ± 0.6**	+1.2 ± 0.7^#^	−0.3 ± 0.6
% change	−1.5 ± 0.6*	+1.2 ± 0.6^#^	−0.2 ± 0.5
Diastolic BP, mmHg	+0.1 ± 0.4	+1.6 ± 0.6**^@^	+0.8 ± 0.4
% change	+0.2 ± 0.6	+2.2 ± 0.8**^@^	+1.2 ± 0.6*
Stroke volume, mL	+1.8 ± 0.7*	+1.2 ± 0.7	+1.5 ± 0.7*
% change	+2.1 ± 0.8*	+1.4 ± 0.9	+1.7 ± 0.8*
Double product, mmHg*beats*min^−1^	−353 ± 76***	−63 ± 99^#^	−208 ± 82*
% change	−4.8 ± 1.0***	−0.1 ± 1.3^#^	−2.5 ± 1.1*
Cardiac output, L*min^−1^	−0.08 ± 0.04	−0.02 ± 0.04^*¦*^	−0.05 ± 0.04
% change	−1.5 ± 0.7*	−0.3 ± 0.8^¦^	−0.9 ± 0.7
Total peripheral resistance, mmHg*min*L^−1^	+0.2 ± 0.1	+0.4 ± 0.2*	+0.3 ± 0.1*
% change	+1.2 ± 0.8	+2.3 ± 0.9*	+1.7 ± 0.8*
Index of contractility, 1000*s^−2^	−0.3 ± 0.6	−0.1 ± 0.6	−0.2 ± 0.6
% change	−0.7 ± 1.0	−0.4 ± 1.1	−0.6 ± 1.0
HF_RRI_LN, ln ms^2^	+0.4 ± 0.1***	+0.2 ± 0.1**^#^	+0.3 ± 0.1***
% change	+6.6 ± 1.1***	+3.6 ± 1.2**^#^	+5.1 ± 1.1***
Baroreflex sensitivity, ms*mmHg^−1^	+1.1 ± 0.7	+2.0 ± 1.2	+1.5 ± 0.9
% change	+6.5 ± 2.3**	+11.3 ± 3.7***^@^	+8.9 ± 3.0***

Values are means ± SEM (standard error of the mean); BP: blood pressure; HF-RRI_LN: high frequency component of RR-interval; ln: natural logarithm; Δ 0–30 min, Δ 30–60 min, and Δ 0–60 min: 0 minutes denotes four minutes after the water drink with calculating a subsequent average over the following 30 minutes, 30 to 60 minutes, and 0 to 60 minutes post-drink, respectively, which were subtracted from each baseline level and presented as deltas. * *P* < 0.05, *P* < 0.01, and ***P < 0.005 statistical significant differences using a paired student’s *t*-Test, where each post-drink period was compared to its baseline values. ^***¦***^ *P* < 0.05, ^@^ *P* < 0.01, and ^#^ *P* < 0.005 statistical significant differences using a paired student’s *t*-Test comparing the changes over 0–30 min with the changes over 30–60 min post-drink.

## Results

Subject baseline anthropometric- and cardiovascular parameters are presented separated by gender and combined in Table 1. When comparing men and women, men were significantly older, taller and heavier than women. Significant baseline gender differences were observed for systolic BP (*P* < 0.005), diastolic BP (*P* < 0.005), total peripheral resistance (*P* < 0.05), and index of contractility (*P* < 0.005; Table 1).

### Haemodynamic changes for men and women combined

The time course (left panels, presented as absolute changes relative to baseline levels) and changes of 0–30 min, 30–60 min, and 0–60 min averages (right panels, presented as percentage change relative to baseline levels) of cardiovascular parameters are shown in Figs 1–4A–F for heart rate, systolic BP, diastolic BP, rate-pressure double product, stroke volume, cardiac output, total peripheral resistance, index of contractility, HF_RRI_LN, and Baroreflex sensitivity separated by gender and combined. Values for each parameter, presented as the change (Δ) from baseline, for all time points are presented in Table 2. In response to the water drink, heart rate and rate pressure double product decreased significantly in the first 30 min compared to baseline (*P* < 0.005 both; Fig. 1A and C). Systolic BP decreased significantly within the first 30 min post-drink (*P* < 0.05; Fig. 1B), but returned towards baseline thereafter resulting in no significant change over the 0–60 min period. Stroke volume increased significantly within the first 30 min post-drink (*P* < 0.05; Fig. 2A) and over the entire 0–60 min period compared to baseline (*P* < 0.05; Fig. 2A). Total peripheral resistance increased significantly from 30–60 min (*P* < 0.05; Fig. 2C) and over the entire measurement period (0–60 min; *P* < 0.05) when compared to baseline.

When results are averaged over time, and values from 0–30 min were compared with those of 30–60 min, significant differences were found for heart rate (−2.2 vs. −1.1 beats*min^−1^; *P* < 0.01), systolic BP (−1.7 vs. +1.2 mmHg; *P* < 0.005), diastolic BP (+0.1 vs. +1.6 mmHg; *P* < 0.01), rate pressure double product (−353 vs. −63 mmHg* beats*min^−1^; *P* < 0.005), and cardiac output (−0.08 vs. −0.02 L*min^−1^; *P* < 0.05) (Table 2, second column).

### Autonomic changes for men and women combined

In response to the water drink, HF_RRI_LN increased significantly within the first 30 min compared to baseline levels (+0.4 ln ms^2^; *P* < 0.005; Table 2, first column; Fig. 3A). The increase in HF_RRI_LN remained significant from 30–60 min post-drink (+0.2 ln ms^2^; *P* < 0.01; Table 2, second column) and over the entire measurement period (0–60 min post-drink) (+0.3 ln ms^2^; *P* < 0.005; Table 2, third column). Despite increases in baroreflex sensitivity of 6.5% (0–30 min; *P* < 0.01; Table 2, first column) and 11.3% (30–60 min; *P* < 0.005; Table 2, second column), no significant change in absolute baroreflex sensitivity was observed in response to water ingestion (Table 2, second column; Fig. 3B).

### Gender effect

Given the baseline gender differences in a number of parameters (systolic BP, diastolic BP, total peripheral resistance, and index of contractility), the percentage change for all parameters was calculated over each time point in order to examine whether gender differences existed, while taking baseline differences into account. Water ingestion induced significant changes in heart rate, rate pressure double product, stroke volume, HF_RRI_LN and baroreflex sensitivity compared to baseline in women. Significant changes in systolic BP, diastolic BP, heart rate, rate pressure double product, HF_RRI_LN and baroreflex sensitivity were observed compared to baseline levels in men (Figs 1–4; right panels, respectively). However, when responses over each time period were compared between women and men, aside from a trend towards significance for stroke volume (Fig. 2, panel D) and index of contractility (Fig. 4, panel D), no significant gender differences (all *P* > 0.10) were identified in response to water ingestion.

## Discussion

In the present study, we aimed to determine in healthy adults a potential cardio protective effect of water. In doing so, we investigated hemodynamic parameters, which were associated with cardiac workload, i.e. heart rate, systolic BP, and rate pressure double product, by using beat-to-beat measurement equipment in order to provide a high time resolution. Additionally, we investigated potential gender related influences on the studied variables. Based on our study results, we provide evidence that drinking water impacts on cardiac workload in a time-dependent fashion whilst these responses appear not to be influenced by gender.

In the current study, we observed an acute and temporary decrease in heart rate, systolic BP and rate pressure double product in response to drinking water, thereby affecting cardiac workload in a time-dependent fashion. These findings corroborate previous reports of decreased heart rate^4, 6, 19, 27^ and decreased rate-pressure double product^4^ in response to water ingestion, which have been investigated in healthy adults. Our observed decrease in heart rate was accompanied by a concurrent increase in the high-frequency power of RR-interval, which is considered as an indicator of cardiac vagal tone at resting conditions^28^, therefore providing evidence for a potential role of the parasympathetic branch of the autonomic nervous system to be, at least partly, responsible for the observed changes in heart rate. Moreover, these findings are in agreement with previous work from another group^6^. A further indication of the involvement of cardiac vagal tone in our observed heart rate responses can be derived from changes in the baroreflex sensitivity, which were similar compared to a previous study where a larger water volume (500 mL) has been used^4^. Indeed, percentage changes in baroreflex sensitivity significantly increased within the first 30 min and thereafter up to 60 min post-drink. One potential biomolecular candidate for linking water ingestion to an activation of vagal tone could be a submember of the transient receptor potential channel (TRP) family. In this context, TRPV4 is capable of sensing and conveying intestinal information on temperature^29, 30^ and osmolality^31^ and, therefore, could be the link between the intestinal absorbed water and an activation of vagal tone.

In the first 10 mins following water ingestion, we observed a pronounced drop in systolic BP of ~−5 mmHg, which was followed by a quick return back toward baseline values. This observation is line with previous studies in healthy young subjects^2, 4^, however, other studies which also used beat-to-beat BP equipment showed no change in systolic BP in response to water ingestion within this period^6, 7^. One possible explanation for this difference could be due to the body position of the study subject, where drops in systolic BP were found in a sitting position^2, 4^, but not in a semi-supine position^6, 7^. A recent review^32^, where potential cardiovascular mechanisms in response to energy drink consumption have been discussed, offered a similar explanation for incongruent hemodynamic findings with regard to caffeinated beverages^32^. As an explanation for the minimal or absent pressor response to water ingestion, it has been suggested that any possible change in BP may be attenuated or even abolished by an increase in cardio vagal drive^6^, thereby counteracting the effects of elevated sympathetic activation observed following water ingestion^5, 7^. On the one hand, and similar to previous studies^2, 4^, the drop in systolic BP in the current study was accompanied by a significant decrease in heart rate and increase in HF_RRI, which suggests that cardiac vagal tone was affected by water ingestion. On the other hand, we observed a small, but significant increase in total peripheral resistance over time, which could be an indirect indicator for an increased sympathetic vasomotor activity following water ingestion.

Similar findings of decreased cardiac workload have been disputed in the past on the basis that increases in stroke volume were observed, which could be an indication for an increase in cardiac contraction force^17, 18^. Although we observed a small increase in stroke volume (+2.9% peak response), the drop in heart rate was greater (−4.5% nadir), thus providing a possible explanation for the declining trend in cardiac output. Here it is pertinent to take the time course of the responses into consideration. In this context, we observed time-dependent reductions in heart rate and systolic BP within the first 30 min post-drink, which in combination with a lower rate pressure double product (a marker for cardiac oxygen consumption)^33^, are thus consistent with a diminished cardiac workload within this period^17^. This concept is further strengthened by our results regarding the index of contractility, a parameter for myocardial contractility^26^, where no changes were observed throughout the test.

Aside from a trend towards gender differences with respect to stroke volume, we were unable to find significant gender differences in cardiovascular parameters in response to water ingestion. To the best of our knowledge, the study by Mendonca *et al*.^19^ is the only study to report gender effects for diastolic BP in response to water ingestion. A striking difference between our study and that of Mendonca *et al*. is that blood pressure was measured intermittently^19^, as opposed to in our study, where BP was measured continuously and beat-to-beat, which could represent a potential limitation of their study.

The current study has a number of practical implications: firstly, we observed a decrease in cardiac workload in response to water ingestion. This may have implications for patients suffering from mild chronic heart failure, i.e. stage A according to the introduced classification from the *American College of Cardiology*/*American Heart Association Task Force* ^34^, where fluids that do not increase cardiac workload could be potentially beneficial given that fluid intake, even if it is restricted, is necessary. Future work is required to investigate the impact of water ingestion on cardiac workload in these patients. Secondly, given that the effect of water on short-term BP in healthy young subjects is currently under debate, this study provides additional data to support the minor pressor effect of water and substantiates existing literature^4–7^. It may be argued that the pressor effect of water is observed in the treatment hypotension associated with, for example, blood donation, prolonged orthostatism and post-exercise recovery in healthy subjects. However, this evidence is heterogeneous and is based on results from single case reports^35^, studies involving individuals at high-risk for syncope^36^, individuals undergoing head-up tilt challenges^37, 38^, or studies which did not measure BP at all^39, 40^. Considering these caveats, as well as the growing body of literature supporting the minor pressor effect of water, future research among young, healthy adults is required in order to elucidate the effect of water in such cases of hypotension.

Caveats of the current study include the retrospective nature in which data were analyzed. Nevertheless, the two original studies were strictly controlled with similar protocols and measurement time points, and given there were no significant differences between the study populations’ anthropometric variables, including cardiovascular baseline parameters, it was deemed appropriate to combine the data. Combining these two studies provided a larger sample size, which allowed for more robust statistical analysis to be carried out. Furthermore, all primary measurement outcome parameters have been derived on a beat-to-beat basis, thereby providing a high time-resolution. A second limitation is the lack of a no-drink/sham control, as the initial studies were designed to measure differences between treatment (energy drink) and control (water). It could be suggested that the effects observed were the result of not only water ingestion, but also perhaps a psychological effect caused by the interruption of the study for the time required to drink the water (4 min). Indeed, normal BP variation has been observed at rest in response to various physiological and emotional stimuli^41, 42^; however, given the retrospective nature of this study, it was not possible to make a comparison between the observed BP changes and normal BP variation. We acknowledge these limitations and agree that future studies investigating cardiovascular responses to water should make an effort to include a no-drink/ sham control and also to compare results with normal variation in measured parameters.

In conclusion, the current study provide evidence that drinking water decreases, in a time-dependent fashion, cardiac workload and that these responses appear not to be influenced by gender. This observed potential cardio protective effect of drinking water seems to be limited for up to 60 min after the drink. Further studies are warranted to address a potential dose-response effect in order to assess an “*optimal*” water load, which could be used in a repetitive fashion over a longer study period to possibly extend the short-term cardio protective nature of water drinking.
